# Epigenetic Dynamics in the Function of T-Lineage Regulatory Factor Bcl11b

**DOI:** 10.3389/fimmu.2021.669498

**Published:** 2021-04-14

**Authors:** Tom Sidwell, Ellen V. Rothenberg

**Affiliations:** Division of Biology & Biological Engineering, California Institute of Technology, Pasadena, CA, United States

**Keywords:** transcription factor, T cell development, context-dependent gene regulation, developmental lineage choice, Runx, chromatin state modulation, repression, chromatin looping

## Abstract

The transcription factor Bcl11b is critically required to support the development of diverse cell types, including T lymphocytes, type 2 innate lymphoid cells, neurons, craniofacial mesenchyme and keratinocytes. Although in T cell development its onset of expression is tightly linked to T-lymphoid lineage commitment, the Bcl11b protein in fact regulates substantially different sets of genes in different lymphocyte populations, playing strongly context-dependent roles. Somewhat unusually for lineage-defining transcription factors with site-specific DNA binding activity, much of the reported chromatin binding of Bcl11b appears to be indirect, or guided in large part by interactions with other transcription factors. We describe evidence suggesting that a further way in which Bcl11b exerts such distinct stage-dependent functions is by nucleating changes in regional suites of epigenetic modifications through recruitment of multiple families of chromatin-modifying enzyme complexes. Herein we explore what is - and what remains to be - understood of the roles of Bcl11b, its cofactors, and how it modifies the epigenetic state of the cell to enforce its diverse set of context-specific transcriptional and developmental programs.

## Introduction

The paralogous Bcl11 zinc finger transcription factors, Bcl11a and Bcl11b, were first identified in a yeast two-hybrid screen for binding partners of the COUP-TF family of nuclear hormone receptors (1). A third related zinc finger factor, Zfp296 (ZNF296 in human), also exists but is not strongly expressed in hematopoietic cells (2). Bcl11a and Bcl11b were subsequently found to be involved in malignancy (3) and ontogeny (4, 5) of the lymphoid system. Despite considerable similarities in their structure (3, 6) and binding sites as defined by *in vitro* biochemical assays (6, 7), distinct roles of the two factors have become apparent in lymphocyte development in the years since. Bcl11a supports the ontogeny of the lymphoid system as a whole (4), as well as the development of B cells (8) and dendritic cell subsets (9); it is also a repressor used to enforce fetal to adult globin switching in erythroid cells (7). In contrast, among leukocytes, Bcl11b has been found to be expressed almost exclusively in the T cell lineage, where it is critical for initial pro-T commitment (10–12), for further developmental choices and correct responses to developmental checkpoints (13–15), and for the stability of mature effectors (16–18). In its only other known hematopoietic role, it is also required for the development and persistence of type 2 innate lymphoid cells (ILC2), an innate immune lineage that does not use T or B cell receptor gene rearrangement but is otherwise functionally closely related to T cells (19–21). Whereas Bcl11a is implicated in malignancy, Bcl11b has primarily been found to act as a dose-dependent, haploinsufficient tumor suppressor (22, 23), suggesting that these related factors can mediate divergent functions that are not exclusively mediated by their shared DNA binding specificity.

Bcl11b is additionally critically required in a number of non-hematopoietic tissues in which it is also specifically expressed, with germline Bcl11b deficiency resulting in perinatal lethality (5). In the central nervous system, Bcl11b regulates appropriate differentiation, migration and function of neural cells, with a number of *BCL11B* mutations resulting in intellectual impairment in humans [reviewed in (24)]. Bcl11b expression is additionally required for normal cranial and tooth development (25–28), mammary tissue, and white adipose tissue (29, 30). In the skin, Bcl11b expression is required for keratinocytes to differentiate appropriately, and to establish and maintain epidermal barrier function (31). With such a diverse array of tissues in which Bcl11b is expressed and acting, the nature of Bcl11b functional specificity is an important question. To fully appreciate its regulation and function, we will argue that Bcl11b function must be considered, at least in part, through the lens of chromatin architecture.

## Bcl11b Structure, Function, and Potential Mechanisms of Action

### Bcl11b Structural Aspects

#### Bcl11b Structure and Protein-Protein Interactions

**Figure 1:**
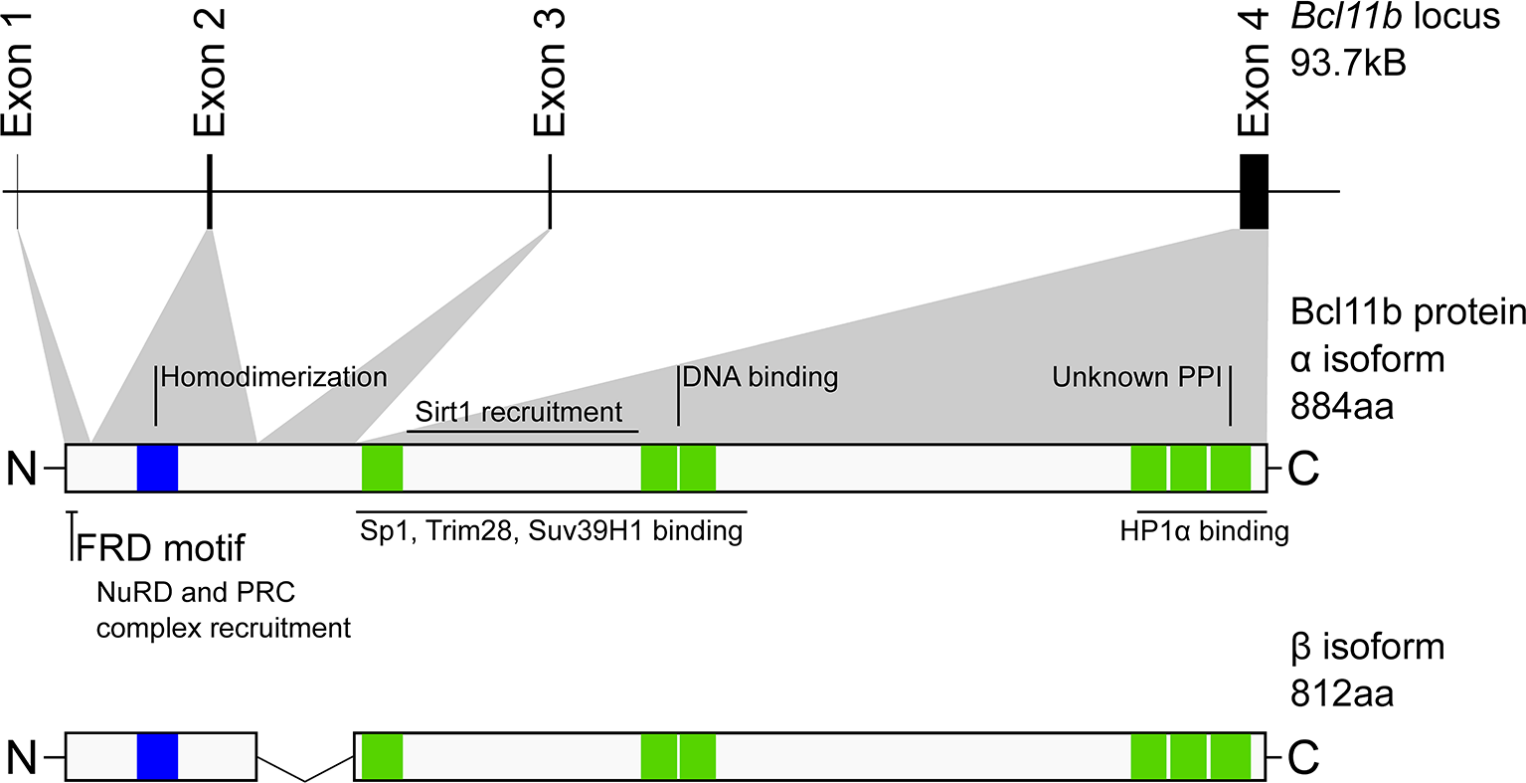
Structure and organization of the murine Bcl11b locus and protein. Scale representation of the distribution of *Bcl11b* exons (upper) and their contribution to Bcl11b protein primary structure (middle). Indicated against this are regions experimentally determined to be involved in cofactor binding or required for Bcl11b function. Lower, Bcl11b β isoform. Not shown is the γ isoform (formed from exons 1 and 4) which is predominantly identified in transformed cells. Blue – C_2_HC zinc finger domain, Green – C_2_H_2_ zinc finger domains. NuRD, Nucleosome remodeling deacetylase complex; PRC, polycomb repressor complex; FRD, FOG repressor domain; PPI protein-protein interaction.

The murine *Bcl11b* gene contains four exons, producing two isoforms by alternative splicing: the full-length (884 amino acids) α isoform, and the 812 amino acid β isoform which lacks exon 3 (**Figure 1**) (32). There are seven zinc finger domains in Bcl11b, all but one C_2_H_2_ type (green in **Figure 1**). A single N-terminal C_2_HC zinc finger domain (blue in **Figure 1**) mediates Bcl11b homodimerization, which is implicated in its transcriptional regulatory activity (33). Intriguingly, the equivalent region in Bcl11a differs by only a single amino acid, and Bcl11a peptides were identified in pulldown experiments using the Bcl11b region as target, raising the possibility that the Bcl11 family proteins heterodimerize through this conserved C_2_HC zinc finger (33). A proline-rich region is found between the first and second C_2_H_2_ zinc fingers and is necessary for SIRT1 recruitment (34, 35). The pair of C_2_H_2_ type Kruppel-like zinc fingers near the middle of the primary sequence mediates DNA binding to the Bcl11b GGCCGAGAGG motif first identified by affinity selection from an oligonucleotide pool (6), or to a pair of alternative motifs, TGACCA or TNCGGCCA, identified by protein binding microarrays (7). Notably, a single amino acid change occurring spontaneously in one of these central zinc fingers in a human patient (N441K) manifested a dominant negative phenotype with catastrophic pleiotropic consequences even for the heterozygote, evidently due to its dimerization with the wildtype protein (27). Finally, there is a cluster of three C_2_H_2_ type zinc fingers within the most C-terminal 100 amino acids of Bcl11b which is also functionally important. The distal-most zinc finger likely mediates as-yet-unspecified but critical protein-protein interactions; mutant protein absent a complete C-terminal zinc finger domain recapitulates the embryonic-perinatal lethality of Bcl11b deficiency and displays hypomorphic Bcl11b activity in various contexts. However, this truncated form surprisingly supports early T cell development and development of ILC2 innate lymphoid cells as long as the host animals remain alive (15, 36). A single amino acid mutation in the penultimate zinc finger (S826G in the mouse), also shows more subtly, though detectably, reduced function (23). Thus, the C terminal fingers appear to be critical for a specific subset of Bcl11b roles, potentially *via* interaction with a unique set of interaction partners. Finally, Bcl11b is subject to numerous post-translational modifications, which can vary by cell type (37) and which can differentially affect positive and negative gene regulation (38, 39). These modifications remain to be studied in depth for their effects on specific partner interactions (discussed below) or on specific developmental functions.

#### Site-Specific Versus Recruited Roles for Bcl11b

Conditional inactivation of *Bcl11b* has strong and specific effects on gene expression in early T cell development (40), mature T cells (41–43) and ILC2 cells alike (19, 37), as described in detail below. These observations indicate that Bcl11b has potent, required, ongoing roles both in activating and in repressing distinctive sets of target genes in these cell types. However, the genes regulated by Bcl11b shift not only between different tissues, but between different stages in the development of a single cell type, e.g. immature vs. mature T cells (11, 13, 40, 44); reviewed in (45). While these effects are further amplified through the operation of gene regulatory network connections downstream of direct Bcl11b targets (14, 40, 44, 46), at least some of these effects are likely to be changes in direct regulation.

One criterion for considering regulation to be direct is binding of Bcl11b to a target gene’s regulatory elements. Several groups have reported Bcl11b ChIP-seq datasets for immature DN thymocytes, DP thymocytes, and mature peripheral T cells (15, 18, 40–44); however, some caveats should be noted. In many regions associated with functionally regulated targets, Bcl11b ChIP-seq conditions used to date can yield low signal-to-noise ratios, and the exact conditions of crosslinking and chromatin fragmentation can affect recovery and relative peak heights. Our own group has been able to obtain robust data from DN pro-T cells by using protein-protein crosslinkers along with formaldehyde, but this may increase the fraction of binding scored that is indirect. Others have obtained strong results from more mature cells with formaldehyde alone, but using micrococcal nuclease rather than sonication for chromatin fragmentation (18, 42). In either case, Bcl11b shows more consistent peak patterns around the target genes that it positively regulates than around its more numerous repression targets. Thus, further possible technical issues, such as under-recovery of compacted, repressed chromatin, may still be obscuring parts of the machinery through which Bcl11b regulates its targets.

The mechanism by which Bcl11b selects its genomic binding sites remains uncertain. Despite the identification of a DNA binding domain in Bcl11b that selectively interacts with a small set of putative Bcl11b target motifs by *in vitro* biochemical assays, ChIP-seq data do not show that Bcl11b prefers binding to genomic sites with these motifs *in vivo*. The most commonly reported motifs enriched by Bcl11b ChIP-seq in T-lineage cells, regardless of the ChIP conditions used, belong to Runx and ETS family members (15, 18, 40, 42, 44). These motifs are identified at high frequency, with – taking pro-T cells as an example – Runx and ETS motifs identified at 44.8% and 36.5% of Bcl11b peaks, respectively (40). This may be based in part on direct protein-protein association; in proteomic analyses of Bcl11b-associating proteins in early T-lineage cells, Runx1 is particularly highly enriched (40). The preference for Runx and ETS motif-containing sites is conserved through lymphocyte development, from pro-T cells to effector CD4^+^ T cell subsets (15, 18, 37, 40, 42–44), and between body systems, being reported in a neural cell line too (47). Runx1 was found to be co-binding at a majority of Bcl11b occupancy sites in pro-T cells, and, genome-wide, target genes that were functionally regulated by Bcl11b in pro-T cells were most highly enriched for sites where the ability of Runx1 to bind stably depended on the presence of Bcl11b as well (40). This suggests that a Bcl11b-Runx1 complex mediates a substantial fraction of the Bcl11b function in these cells, though the specific order in which these factors tend to be recruited remains to be established.

Despite the similarity of the motifs bound, Bcl11b does not bind to the same genomic sites in different developmental contexts, or even in different T-lineage developmental stages, and this will be important to note throughout the following sections. Naïve CD4 T cells show different patterns of Bcl11b binding around Treg-lineage genes than do Treg cells (43). Both ILC2 cells and DN3 pro-T cells require Bcl11b for their generation and function, but there are more lineage-specific than common Bcl11b binding sites identified when comparing Bcl11b ChIP-seq from ILC2 and DN3 cells (37). In part, this conditional choice of occupancy sites is likely to reflect interactions with different binding partners. In Th2 cells, a GATA3-high T cell lineage, many of the Bcl11b occupied sites are also linked to GATA motifs (41), whereas in similarly GATA3-expressing ILC2 cells Bcl11b binding is found at sites enriched for BATF/JunB type basic leucine-zipper (bZIP) motifs. Indeed, whereas proteomic analyses show Bcl11b protein binding strongly to Runx family factors as well as to GATA3 in T lineage cells, it has a different set of preferred binding partners in ILC2 cells (37, 41). The large difference between the *in vivo*-enriched target motifs and the *in vitro* defined Bcl11 family motifs, and their strong context dependence, make a compelling argument that much of the selectivity of Bcl11b deployment across the genome is based on recruitment stabilized by protein-protein interactions rather than by direct recognition of versions of a core DNA sequence.

### Diverse Impacts of Bcl11b on T-Cell Development

#### Direct and Indirect Bcl11b Effects on Gene Expression During Early T-Lineage Commitment

**Figure 2:**
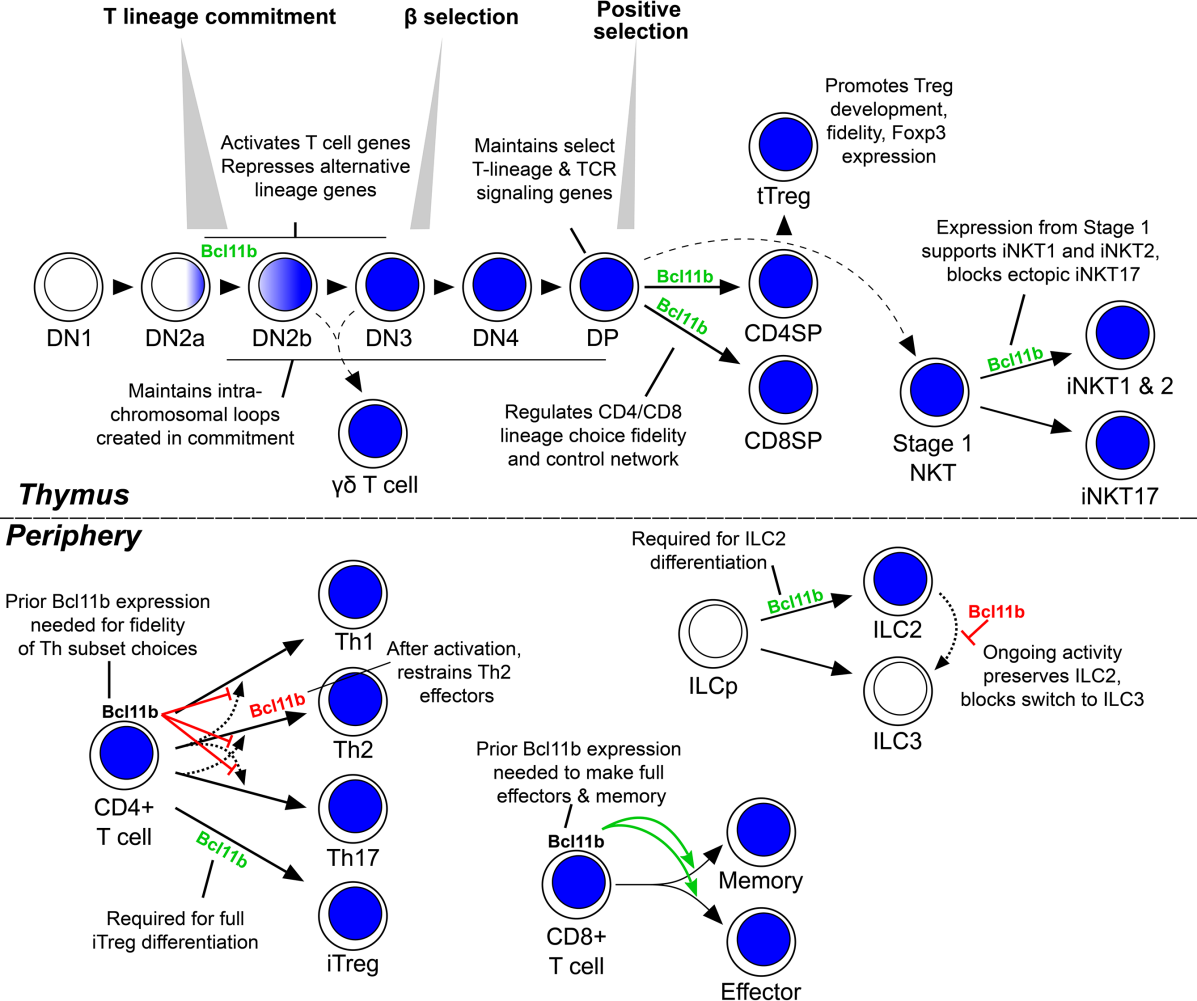
Roles of Bcl11b through lymphocyte development and differentiation. Schematic showing the developmental relationships of various lymphocyte lineages. Blue shading indicates Bcl11b-expressing developmental stages. Green text and arrows indicate supportive roles of Bcl11b is the indicated process, red text and flat arrows indicate an inhibitory role. Not shown is the NK-like population that the various DN2b-onward thymic stages may differentiate into following Bcl11b removal. DN, double negative; DP, double positive; SP, single positive; tTreg, thymic regulatory T cell; iNKT, invariant natural killer T cell; Th, helper T cell; iTreg, inducible regulatory T cell; ILC, innate lymphoid cell; ILCp, ILC precursor.

The impacts of Bcl11b on T cell development are exerted throughout all phases of the T lineage from the first stage when Bcl11b is expressed (**Figure 2**). Work to date in the mouse has identified Bcl11b to be necessary to enforce T lineage commitment, from the time of its first expression (10–12). The earliest stages of T cell development, identified by a CD4 and CD8 double negative (DN) surface phenotype, can be further divided into discrete, sequential stages denoted DN1 to DN4. Stages DN1–DN3, prior to the first T cell receptor (TCR) gene expression, are also referred to as pro-T cells. Through the first stages of development in the mouse thymus, precursors of the future T cells maintain a conditional multilineage potential, which is held in check by Notch pathway signalling while they are embarking on the T cell program. By the middle of the DN2 stages, the cells undergo commitment so that they lose their intrinsic capacity to adopt alternative fates (48). *Bcl11b* expression begins in the DN2 compartment, during the transition from DN2a cells to DN2b cells, distinguishable by degree of c-Kit protein expression. A *Bcl11b* gene expression-linked fluorescent reporter protein has shown that individual cells that have activated *Bcl11b* expression have given up their alternative potentials and have undergone commitment (49), and this is to date the best single marker available to score the commitment transition. In human pro-T cell development, single-cell transcriptome analysis suggests that *BCL11B* is upregulated at a corresponding time (50, 51). This Bcl11b expression is required to promote expression of T lineage genes and restrict access to alternative developmental lineages (10–12), as well as to permit TCRβ expression and to make development of αβ T cells possible at all (5). Fetally-derived T cell precursors lacking Bcl11b are not only developmentally blocked, but also show abnormally prolonged access to myeloid and dendritic-cell fates as well as to natural killer (NK) fates (10, 12), whereas postnatal cells lacking Bcl11b show NK-like or innate lymphoid cell-like features (11, 40). Meanwhile, Bcl11b gain-of-function appears to exert a positive effect on cell-surface markers of T-cell developmental progression in human pro-T cells (52) and to provide partial developmental rescue of mouse pro-T cells lacking TCF1 (53).

**Figure 3:**
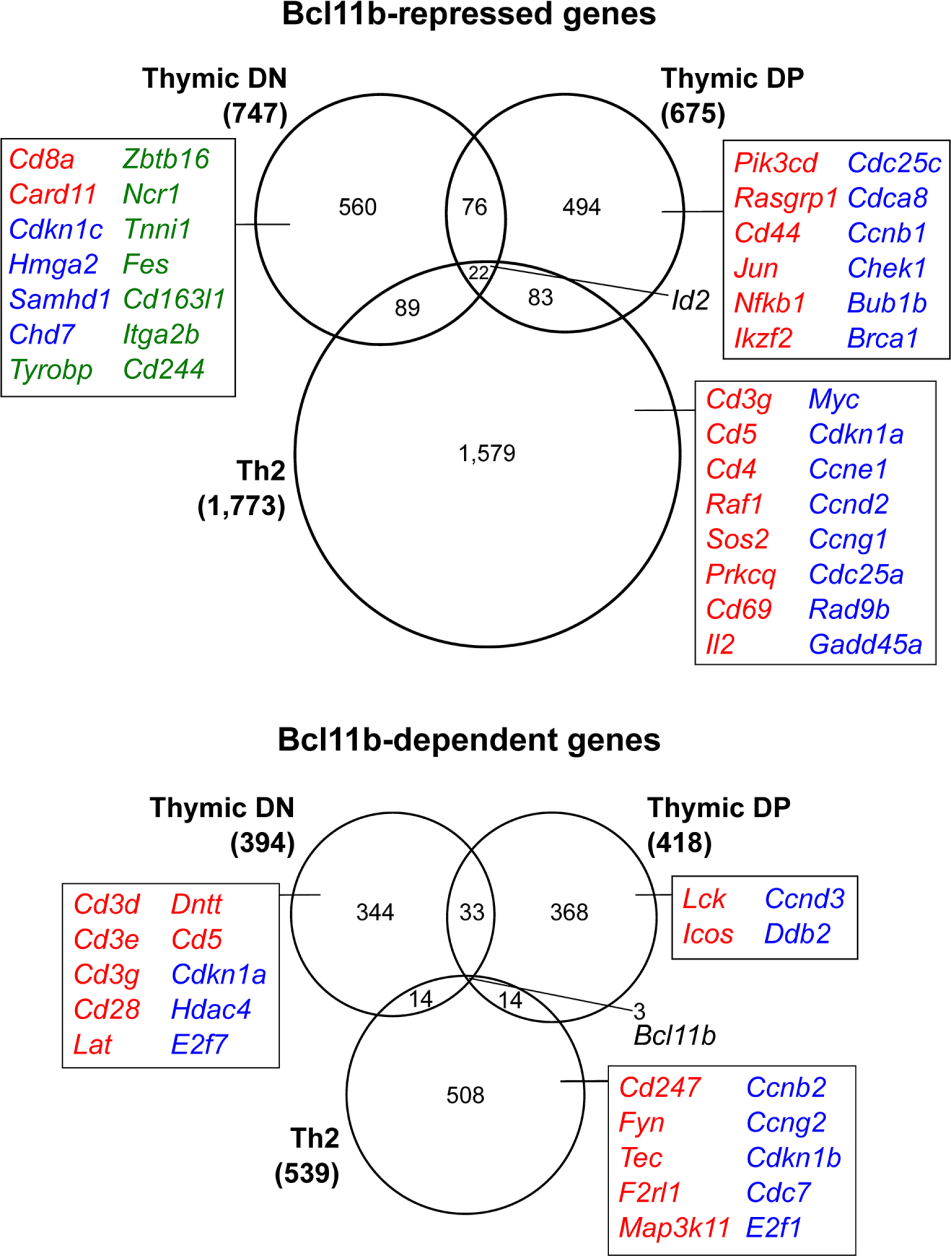
Context-dependent transcriptional regulation by Bcl11b. Genes differentially expressed in the absence of Bcl11b in thymic double negative cells (40), double positive cells (14) and Th2-polarized mature CD4 T cells (41). Selected genes involved in T cell receptor signaling (red), regulation of cell cycle (blue) or alternative lineage differentiation (green) are highlighted to exemplify processes that display varying Bcl11b-dependent regulation in developmentally distinct cellular contexts. Gene lists extracted from Table S3 of reference (40) (‘high confidence’ targets identified as differentially expressed in at least two of the three Bcl11b deletion models), Table S1 of reference (14) and Table S1 of reference (41).

Patterns of gene expression changes during these early stages of mouse T cell development have been characterized with increasing comprehensiveness (48, 54–59), and one might speculate that Bcl11b may repress some regulatory factor(s) that promote multipotency before commitment, and subsequently initiate expression of T cell identity genes that operate after commitment. Indeed, some T-lineage genes are turned on in a Bcl11b-dependent manner at the DN2b stage, such as those encoding the TCR signalling components CD3-γ, -δ and -ε, terminal deoxynucleotidyl transferase (*Dntt*), CD5, and gene products unique to the DN3 stage, which follows shortly after *Bcl11b* activation (**Figure 3**) (40, 44). Even more genes appear to be repressed by Bcl11b in these early T-lineage cells, genes that are upregulated greatly in *Bcl11b* knockout pro-T cells. Bcl11b appears to repress the stem cell growth factor receptor gene *Kit* directly, but its repression targets for the most part do not encode stem and progenitor-associated “master multipotency” regulators. Instead, they encode a wide variety of signalling molecules, receptors, and transcription factors used in specific alternative hematopoietic programs, some NK-affiliated, some myeloid-like, some used in B cells, and some typical of γδ T cells (**Figure 3**, green text). These genes are potentially direct targets of Bcl11b repression, and may serve as useful examples for analysis of Bcl11b regulatory mechanisms, in light of the prevalence of repression-associated protein complexes among Bcl11b interaction partners (discussed in final section).

The entire TCRβ locus may be among the direct targets of Bcl11b-complex action. It was noted very early that *Bcl11b* mutation completely blocked αβ T cell development from the β-selection checkpoint, but spared at least some lineages of *γ*δ T cells (5). This result has been reconfirmed and extended repeatedly (12, 14, 60, 61) and has been shown to be functionally important for the human αβ vs. γδ T cell lineage decision as well (62). As the two lineages share a requirement for at least some of the genes responding to Bcl11b in pro-T cells - such as the CD3 coreceptors - this suggests that there may be poorly understood differences in the ways these shared, pan-T genes are regulated in these two early-separating branches of T cell development.

*Bcl11b* disruption can affect more genes beyond direct targets of Bcl11b, of course. In human pro-T cells BCL11B is needed to enable cells to exit from the precommitment state, by a mechanism that closes chromatin at sites associated with the progenitor-associated transcription factor PU.1 (63). In the mouse, one central regulator that appears to play a significant gene network role downstream of *Bcl11b* removal is Id2, an E protein transcription factor antagonist. *Id2* is normally under repression by Bcl11b within mouse DN and later thymocytes and T cells (11, 12, 14, 40, 41, 44). However, a key subset of *Bcl11b* perturbation effects in mouse pro-T cells turn out to depend on *Id2* (40), and could thus be mediated indirectly, as Bcl11b protects E proteins from Id2-mediated inhibition. *Id2* is not normally expressed in pro-T cells before they turn on *Bcl11b* and does not represent a stem-ness function as such, but it is an important divider of the innate lymphoid cell fates from the T and B cell fates (64–68). Thus, in this context Bcl11b’s repression of *Id2* keeps pro-T cells in the adaptive immune cell pathway. Interestingly, though, Bcl11b’s role in repressing this target is highly context dependent. All ILC lineages depend on *Id2* expression, but one, the ILC type 2 lineage, also expresses and depends on Bcl11b as noted earlier (19–21). In these ILC2 cells, Bcl11b does not repress *Id2*, as a consequence of its different genomic DNA binding pattern and different interaction partner associations (37) already noted above. Conversely, in the human, thymocytes may not require *BCL11B* to keep *ID2* silent (52).

#### Bcl11b Regulates Gene Expression, Selection, and Fate Choice of Committed Thymocytes

##### Establishing a Competent DP Thymocyte State

Despite the requirement for Bcl11b to turn on basic components of T cell identity initially, it is not required to maintain their expression. Case in point: disruption of the *Bcl11b* gene prior to its expression produced cells with severely reduced expression of several genes coding for T cell receptor (TCR) signalling molecules, *Cd3e*, *Cd3g* and *Zap70* (12), but expression of these genes was unaffected by deletion later, using *Cd4*-Cre to inactivate *Bcl11b* from the DP stage (14). Alternate suites of TCR-signalling associated molecules required Bcl11b for their expression at the DP stage (**Figure 3**). Pre-positive selection DP cells that lacked Bcl11b showed reduced expression of the *Lck*, *Socs3* and *Sit1* genes (14), while postselection cells showed reduced expression of CD5 and a failure to upregulate the αβ TCR complex itself in the absence of Bcl11b (13). Associated with these gene expression changes, many defects were identified in cells lacking Bcl11b from the DP stage that could interfere globally with their positive selection, with *Bcl11b*-deleting thymocytes found to display impaired TCR signaling at multiple TCR distal readouts (phosphorylation of Zap70, Slp76 and Erk, nuclear NFAT4 import, and calcium flux), and a survival defect independent of TCR signals (13).

##### Bcl11b Preserves Fidelity of the CD4/CD8 Lineage Choice

More recently, Bcl11b has proven to be required in DP thymocytes to allow them to undergo lineage branching subsequent to positive selection and to guide their divergent development into mature CD8 and CD4 single positive (SP) T cells. Choice of SP lineage is linked with positive selection and typically occurs in a developmentally-timed, TCR-dependent manner (**Figure 2**). As positively selecting TCR signals begin, DP cells partially downregulate expression of the CD8 coreceptors that otherwise stabilize TCR interaction with Class I MHC molecules, while preserving the CD4 coreceptors that collaborate with TCR at Class II MHC molecules. A resultant reduction in TCR-coreceptor signals received indicates the cell’s rearranged TCR is optimised to receive signaling through MHC-I, and initiates Runx3-dependent CD8SP lineage commitment. If CD4^+^CD8^int^ cells detect no decrease in TCR-coreceptor signals, this indicates that CD4 is co-engaged with TCR at class II MHC instead. These cells then respond by upregulating ThPOK, resulting in ThPOK-dependent commitment to the CD4SP lineage (reviewed in (69)). This decision normally involves a mutual repression gene network circuit between ThPOK (encoded by *Zbtb7b*) and Runx3, which also divergently regulates *Cd4* and *Cd8a*/*Cd8b* themselves through well-defined cis-regulatory elements (69, 70). Although Bcl11b has been implicated in regulating TCR signal strength in the DP compartment (13), its function in subsequent SP lineage choice is independent of this role. Rather than resulting in preference for one over another SP lineage, Bcl11b loss was shown to cause aberrantly persisting coexpression of both CD4 and CD8, due to coexpression of CD4- and CD8-central lineage regulators ThPOK (*Zbtb7b*) and Runx3, respectively (15, 36). While the CD4^+^CD8^+^ phenotype of these cells could suggest precocious activation of the two effector lineage regulators (14), other markers showed that many or most of the ambiguous cells were actually post-positive selection (identified by a TCRβ^high^CD24^low^ phenotype). Thus, the normal mutual repression circuit governing CD4/CD8 divergence was broken by loss of Bcl11b. In addition, generation of cells with this ‘scrambling’ of lineage choice occurred in either MHC-I or MHC-II deficient host animals, identifying the phenomenon as independent of the typical TCR:MHC-dependent selection process (15).

**Figure 4:**
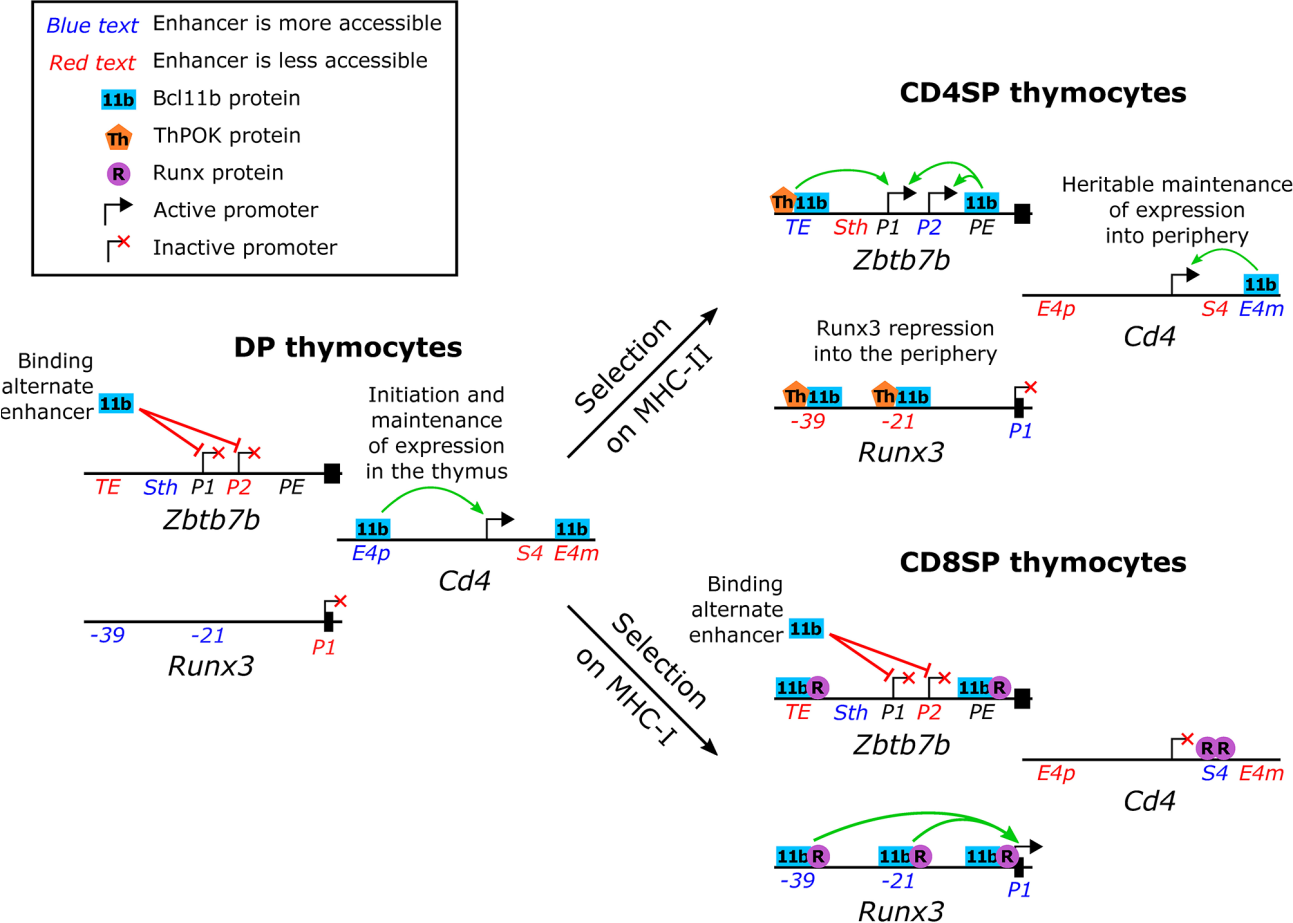
Normal functions of Bcl11b in single positive thymocyte lineage commitment. Schematic representation (not to scale) of the upstream regions of the *Zbtb7b* (ThPOK), *Runx3* and *Cd4* loci in pre-positive selection double positive and in single positive thymocytes. Enhancers that show a dynamic difference in accessibility are indicated in either blue (more accessible than in the comparator thymocyte subsets) or red (less accessible than in the comparator thymocyte subsets) as determined by chromatin modifications [*Cd4* enhancers (36)] or ATAC-seq [*Zbtb7b* and *Runx3* enhancers (2)]. Green and red arrows indicate supportive and inhibitory roles, respectively, of Bcl11b in transcription.

As in the earlier T cell lineage commitment transition, Bcl11b is implicated in both activation and repression of directly-bound target genes in CD4SP/CD8SP lineage choice. The cis-regulatory elements required for these activities have been most intensely studied in this system, and have yielded some surprises. In several respects, Bcl11b works to favor the CD4SP helper fate. First, Bcl11b was found to be needed for appropriate repression of *Runx3*, although its binding did not always result in repression. Two Bcl11b- and Runx-binding elements were identified, (21 and 39kb) upstream of the *Runx3* distal *P1* promoter. While these sites worked redundantly as enhancers supporting expression of a *Runx3* reporter construct normally in CD8SP cells, these sites were able to bind ThPOK as well in a CD4SP context, and this ThPOK binding mediated repression. Notably, ectopically overexpressed ThPOK was only able to downregulate *Runx3* expression in the presence of full-length Bcl11b (15). Thus, Bcl11b could favour the CD4 cell lineage as a required factor supporting ThPOK to enforce repression at key *Runx3* enhancers (**Figure 4**, upper right, “Runx3 repression into the periphery”). Second, Bcl11b was found to bind to the known regulatory elements of the *Cd4* gene, targets where it apparently exerted positive regulatory effects both for activation and for maintenance in the CD4SP lineage (36) (**Figure 4**, upper right).

The pivot of the CD4/CD8 lineage choice, however, is the choice to activate or not to activate Th-POK (*Zbtb7b*) expression, and here Bcl11b’s role becomes more complex (**Figure 4**). Within the CD4SP lineage, Bcl11b also appears to act as a positive regulator for *Zbtb7b*, controlling expression intensity (15). ChIP-seq data from whole thymocytes showed that Bcl11b binds to the *Zbtb7b* locus in a broad pattern of occupancy over at least 20 kb, stretching across the coding sequence and its known regulatory elements. Focused ChIP-PCR showed that Bcl11b bound together with Runx1 at two known regulatory regions. At one enhancer it collaborated with ThPOK as well, and its presence substantially promoted activity from both *Zbtb7b* promoters (15). However, Bcl11b was also required to keep this same *Zbtb7b* locus silent in both preselection DP cells and postselection CD8SP cells. In CD8SP cells, surprisingly, it was found co-binding with Runx factors at the same known regulatory elements as in CD4SP cells, with only modest shifts in relative occupancy, raising the question of how its role had reversed (**Figure 4**, upper and lower right). Interestingly, targeted deletion studies showed that Bcl11b apparently did not depend on these known enhancer or silencer elements for its repression activity in CD8SP cells (15). It remains to be established whether other parts of the Bcl11b binding profile across the *Zbtb7b* locus map another discrete functional element responsible for this silencing activity, or whether Bcl11b’s repression activity is mediated through a different mechanism. However, the results clearly show that Bcl11b can play context (lineage and cofactor) dependent roles in supporting or repressing transcription, even of the same gene. Thus, the observations summarized here place Bcl11b in the middle of the CD4/CD8 effector lineage choice, but with an ambivalent, context-dependent role, supporting the decisiveness of both outcomes.

#### Bcl11b in the Lineage Stability of Mature T Cells

In mature effector T cells, the role of Bcl11b becomes more complex and even more difficult to associate with specific, dedicated target genes (45) (**Figure 2**). The first reports of Bcl11b’s role in lineage fidelity came from the CD8SP cytolytic T cell lineage. In steady state, peripheral CD8 T cells that had previously lost Bcl11b function (by distal Lck-Cre mediated deletion) shortly after intrathymic positive selection were functionally deficient, failing to appropriately expand or upregulate expression of key effector genes in response to antigenic stimulation. This was ascribed in part to defects in maintenance of key TCR signaling molecules (PLCγ1) and activity of directly Bcl11b-regulated *Cd8* locus regulatory elements in the absence of Bcl11b (71, 72). Acute Cre-ERT2-induced inactivation of Bcl11b in mature CD8 T cells resulted in ectopic upregulation of a subset of NK lineage-associated genes despite retention, though at reduced levels, of cell-surface lineage markers CD3 and CD8 (11). This effect appeared to echo the response seen much earlier in development, when Bcl11b was removed from progenitors before TCR expression and DN2/DN3 cells switched into an NK-like developmental program (11). The role of Bcl11b here might be understood as a relatively simple case of keeping NK effector genes under repression and allowing appropriate maintenance of CD8^+^ T-lineage gene expression. However, Bcl11b also has complex roles in the CD4^+^ subsets that require other explanations.

The regulatory T (Treg) cell lineage shows a strong requirement for Bcl11b activity, and the results of Bcl11b removal from Treg cells provide an example of a more general role of Bcl11b in the integrity of specific T-cell programs (**Figure 2**). Inactivation of Bcl11b in all T cells using a *Cd4*-driven Cre resulted in wasting disease, as Bcl11b-deleted Treg cells were reduced in number, reduced in their levels of Foxp3 expression, and they ectopically expressed inflammatory cytokines (16). Even more severely, thymocytes expressing a hypomorphic Bcl11b that lacks the C-terminal zinc finger failed to produce thymic Treg cells at all in a fetal liver-derived chimera model (15). Prior binding of full-length Bcl11b to the *Foxp3* intronic pioneer element CNS3 was required to allow Satb1 to bind (15) to activate the *Foxp3* locus, potentially explaining this dependency. The inducible differentiation of Treg cells *in vitro* from peripheral conventional CD4 T cell precursors (a distinct process from thymic Treg cell differentiation, and meant to mimic differentiation in the gastrointestinal compartment) similarly resulted in suboptimal conversion when the cells were deprived of full length Bcl11b (15, 16). Specific *Bcl11b* inactivation in mature Treg cells, using *Foxp3*-driven Cre-recombinase, also resulted in significant loss of the Treg cell compartment *in vivo* and fatal autoimmunity (43). Most strikingly, analysis of individual *Bcl11b*-deleting cells identified not only loss of expression of core Treg lineage genes, but also ectopic upregulation of NK- and critical myeloid-lineage associated genes, including *Spi1* (encoding PU.1) and *Cebpa* (43). This represents an apparent degradation of the integrity of the whole T lineage program and reversal of T-lineage commitment when Bcl11b is lost in the mature Treg cell context.

Loss of Bcl11b interferes with other CD4 T cell effector functions as well (45, 73), but the results seen depend strongly on the variety of helper T (Th) cell response being favoured by environmental stimulation conditions. To summarize, prior loss of Bcl11b appears to break the expected connections between a type 1, type 2, or type 17 polarization response and the environmental stimuli that normally favor it. *Bcl11b* inactivation specifically in mature T cells, under the control of a distal *Lck* promoter-driven Cre recombinase, has resulted in defective Th subset polarization in any of a variety of directions (**Figure 2**). For example, in the Th17-polarizing model of experimental autoimmune encephalomyelitis, responding Bcl11b-deficient CD4 T cells lost autoimmune effector function and gained expression of Th2 factors GATA3 and IL-4, even while maintaining their levels of Th17 factors (17). In contrast, in the same mouse line, in the Th2-promoting models of *Heligmosomoides polygyrus* infection and house dust mite sensitization, Bcl11b-deleted cells showed decreased Th2 effector activity. Notably, the Bcl11b-deleting CD4 T cells in this system showed reduced expression of Th2 transcripts (*Gata3, Il4*) but upregulation of core Th1 (*Tbx21, Ifng*) and Th17 (*Rorc, Il17a*) transcripts instead, even after preselecting for IL-4 expressing effector cells (18). The loss of GATA3-expressing Th2 cells and increase in RORγt expression in this case is echoed by the effects of acute loss of Bcl11b in type 2 innate lymphoid cells, which resulted in a reduction in the expression of ILC2-associated genes, an upregulation of ILC3 genes, and even protection from an infection model typically controlled by ILC3 cells (19). A thematically similar role for Bcl11b in insulating future effector subset identity choice has also been observed in the special agonist-selected invariant NK-like T (iNKT) cell lineage. In this lineage, loss of Bcl11b activity from an early thymic stage resulted in a sharp reduction in Th1- and Th2-like iNKT cells, with those remaining showing ectopic expression of a number of factors normally associated with the Th17-like iNKT lineage (**Figure 2**) (74). Strikingly though, in a model where T cells first developed in the presence of normal levels of Bcl11b and then were deprived of Bcl11b acutely, the effect on Th2 lineage integrity was reversed. Conditional disruption of *Bcl11b* in peripheral T cells (tamoxifen with Cre-ERT2) at or following T cell activation did not reduce Th2 function; instead, it upregulated Th2 differentiation and repressed alternative Th1 differentiation (41), a distinctly contrasting phenotype to that of prior deletion. These observations highlight an important context dependence for the timing of Bcl11b activity in mature CD4 T cells and their innate counterparts alike.

These observations implicate Bcl11b in maintaining the integrity of lineage identity distinctions between highly developmentally related lymphocyte subsets. Importantly, though, as already seen for regulation of positive targets like the *Cd3* genes (**Figure 3**) or positive/negative targets like *Zbtb7b*, developmental timing of the Bcl11b effect appears to be critical. Inducible (tamoxifen with Cre-ERT2) inactivation of *Bcl11b* after CD8 T cell activation did not recapitulate the defective effector differentiation seen in CD8 T cells that inactivated *Bcl11b* earlier, under control of the distal Lck promoter driven Cre (11, 71). Taken alongside the contrasting observations in studies of Bcl11b in Th2 differentiation, we have diverse contexts in which Bcl11b inactivation following T cell stimulation fails to recapitulate the phenotype of prior inactivation. Such discrepancies seem to indicate a developmental window in between T cell maturation and antigenic challenge in which Bcl11b expression is required to establish the machinery to insulate the integrity of future T cell effector responses. A potential way to account for this long-term, heritable difference in effects could be if Bcl11b is responsible for initiating or maintaining regulation at the level of chromatin architecture.

### Bcl11b and Chromatin Modulation

#### Bcl11b Interaction Partners

An explanation is needed for the extreme context-dependence of Bcl11b binding and activity, even within a single lymphocyte lineage (**Figures 2**, **3**), and the puzzlingly broad impact of knocking down Bcl11b in different effector sets of Th cells, where loss of Bcl11b and the timing of loss of Bcl11b seem to impact each of the divergent effector programs tested (43, 73, 75). The limited information available already suggests that Bcl11b function is strongly influenced by interactions with its transcription factor partners. There are several ways that this could work. Partners could give Bcl11b distinct binding specificities, bringing it to different enhancer sites; partners could give Bcl11b different effector functions, switching between activator, repressor, and placeholder; or Bcl11b might not be working *via* regulating transcription as such at all, but working instead by modulating and maintaining different chromatin architectures that set the playing field for all the other factors in the system. The answers are not clear yet, but biochemical evidence is in hand to weigh specific options.

It is notable that the original identification of Bcl11b – as “CTIP2” – was as a non-DNA binding co-repressor partner of the nuclear receptor factors COUP-TFI and COUP-TFII (Nr2f1 and Nr2f2) (1). Although the Nr2f family itself seems less important for Bcl11b recruitment in lymphocyte biology, the relative significance of direct vs recruited Bcl11b to its varied functions in regulating gene expression in lymphocyte lineages remains to be determined. Direct identification of Bcl11b binding partners by co-immunoprecipitation (co-IP) and mass spectrometry has been carried out in various cell types. As noted above, Bcl11b also interacts strongly with Runx family factors, and a majority of its binding sites in pro-T cells are co-occupied with Runx1 (40). In Th2 cells, it has a prominent interaction with GATA-3 detected both on and off the DNA (41). Such partner preferences mean that availability of the interacting factor can influence the patterns of Bcl11b activity sites in different contexts. However, while no intrinsic enzymatic activity has been identified to date for Bcl11b protein itself, many of its other co-bound cofactors suggest possible roles in modifying chromatin structure and regulating its architecture (**Table 1**), in a manner potentially more comparable to a scaffold protein than a classic transcription factor.

**Table 1:** Chromatin modifying activities of Bcl11b co-bound factors.

Associated factor	Evidence of association	Chromatin architectural changes mediated
NuRD complex	Co-IP followed by western blot (33, 76–78) and mass spec.; ChIP colocalization (40)	HDAC-1 and -2 mediated deacetylation (broad substrate range) CHD/Mi-2 mediate ATP-dependent chromatin remodeling
PRC1	Co-IP followed by western blot and mass spec.; ChIP colocalization (40)	H2AK119 monoubiquitination Establishment and maintenance of TADs
PRC2	Co-IP followed by western blot (77)	H3K27 trimethylation
NRSF complex	Co-IP followed by mass spec.; ChIP colocalization (40)	Sin3 and Rcor proteins recruit HDAC1/2 (deacetylation, broad substrate range) Kdm5a mediated H3K4-tri and -di methyl group removal
Kdm1a/LSD1	Co-IP followed by western blot (79) and mass spec (40).	H3K4me and H3K9me demethylation
SWI/SNF (BAF) complex	Co-IP followed by mass spec (40, 80)., co-sedimentation (80, 81)	Chromatin remodeling TAD formation and insulation
Trim28/KAP1	Co-IP followed by western blot (82), mass spec (40).	SETDB1-mediated H3K9 mono-, di- and tri-methylation. NuRD recruitment.
Sirt1	Western blot of co-IP (34, 35, 77)	H3K9, H3K14, H3K16, H1K26 deacetylation
Suv39H1	Western blot of co-IP (83)	H3K9 trimethylation
P300	Western blot of co-IP (84)	H3K18, H3K27 acetylation Chromatin remodeling

Factors and complexes identified to bind Bcl11b, the evidence supporting the association, and the chromatin modifying activities of each factor/complex. IP, immunoprecipitation; mass spec, mass spectrometry; TAD, topologically associating domain; ChIP, Chromatin immunoprecipitation.

Proteomic assays have also identified a number of co-bound factors with chromatin modifying activities to be high-scoring interaction partners of Bcl11b, even more prominent than sequence-specific transcription factors (33, 40, 76, 85). Partners include multiple members of the NuRD (33, 40, 76), Polycomb Repressive Complex (PRC)-1, REST and Kdm1a complexes (40). Supporting these results for purified protein complexes, pro-T cell ChIP-seq assays showed extensive overlaps in genomic occupancy patterns between Bcl11b and Chd4, Mta2 (NuRD complex), Rnf2 (PRC1 complex), NRSF (RE-1 silencing *REST* complex) and/or Kdm1a (LSD1) (40). Over 90% of the genomic occupancy sites for NuRD complex component Mta2 binding were also associated with Bcl11b binding in pro-T cells, whereas the other factors showed varying degrees of independence of the Bcl11b pattern (40). REST, Kdm1a, and NuRD complexes are traditionally associated with repression, and the mass spectrometric (40) and western blot (82) analyses of co-IPs showed that Bcl11b was also highly associated with Trim28 (KAP-1), a potent co-repressor for KRAB-domain containing zinc finger transcription factors (86, 87). In a different developmental context, moreover, BCL11B transfected into human kidney-derived HEK293T cells showed interactions with members of two other repressive complexes, PRC2 member EZH2 (77) and histone deacetylase SIRT1 (34, 77).

Not all the associations of Bcl11b were necessarily associated with repression. Some members of the switch/sucrose non-fermentable (Swi/Snf) - aka BRG1/BRM-associated factors (BAF) - complex were additionally identified among Bcl11b co-IPs, albeit with lower prevalence than the NuRD complex components (40). This is consistent with work identifying Bcl11b association with Swi/Snf complexes in various non-leukocyte systems (80, 81). In addition, the interaction partners reported in early T lineage cells included three members of the Mediator Complex (40) through which RNA polymerase II at promoters interacts functionally with enhancers (88). Such complexes could mediate positive regulatory roles of Bcl11b, although this remains to be tested. Thus, diverse roles of Bcl11b could emerge from its binding with different cofactors.

The portions of the Bcl11b structure that mediate interactions with these partners (**Figure 1**) remain only partially defined, possibly because the interaction partners are large multiprotein complexes with diverse subunits that could present multiple binding interfaces with Bcl11b. Early work identified the N-terminal portion of Bcl11b to mediate binding to the RBBP4 and RBBP7 (RBAP48 and RBAP46, respectively) proteins, which may direct further recruitment of nucleosome remodeling and deacetylation (NuRD) complex (76) and PRC2 component proteins. Histone deacetylase SIRT1 was also identified early as a BCL11B binding partner, with the region between the first and second C_2_H_2_ zinc fingers (and the equivalent region in BCL11A) sufficient to precipitate SIRT1 *in vitro* (34, 35). The extreme N-terminus of Bcl11b contains an MSRRKQXXP motif, part of the FOG repressor domain (FRD), which is highly conserved in the Bcl11 superfamily of proteins (77, 89). Although Bcl11b truncated forms containing only residues 129-350 (and therefore not the N-terminal MSRRKQXXP motif) could strongly bind RBBP4 and RBBP7 in pulldowns from purified protein mixtures (90), the N terminal FRD has proven to be required for Bcl11b binding to NuRD complexes *in vivo*. In non-lymphocytic cell lines, residues 145-434 of the β isoform were sufficient to precipitate Suv39H1 and Sp1, and residues 717-813 for HP1α (83). Phosphorylation of Serine-2 in Bcl11b, or mutation of this residue, resulted in significantly reduced interaction of Bcl11b with NuRD complex proteins in CD4 T cells (78). Similarly, mutation of the Arginine-3 residue to Serine was sufficient to interrupt recruitment of both NuRD and PRC2 complexes by Bcl11b, which was ascribed to a failure of the mutant domain to bind RBBP4 or RBBP7 (77). This single amino acid mutation yielded a skull seam closure defect in heterozygous humans and perinatal lethality in homozygous mice (77). Notably, a Bcl11b mutant absent the most C-terminal zinc finger is similarly perinatally lethal in the absence of wild type Bcl11b (15), although no interaction partners for this domain have been described.

#### Chromatin-Modulation Roles of Bcl11b Interaction Partners

The complexes and factors that are bound by Bcl11b have well-studied roles in regulating local chromatin modifications, as summarized in **Table 1**. The REST (for Neuron-Restrictive Silencing Factor, NRSF) complex establishes repressive histone marks, through the cofactors recruited by the sequence-specific transcription factor NRSF. Of these, Sin3a and Sin3b and the CoREST factors (Rcor1 and Rcor2) recruit histone deacetylases, while Sin3b-recruited Kdm5a may catalyse removal of transcriptionally activating H3K4 di- and tri-methylation [reviewed in (91)]. The PRC1 and PRC2 complexes are also transcriptional repressors. PRC2 functions to mediate H3K27 trimethylation for facultative repression of target genes. PRC1 can dock to these H3K27me3 marks and monoubiquitinate H2AK119 to allow longer-lasting repression of target genes. Sirt1 is an HDAC which relies upon NAD^+^ for its activity, modulating its activity according to the metabolic state of the cell [reviewed in (92)]. Sirt1 has a broad range of targets, and is capable of deacetylating H3K9, H3K14, H3K16 and H1K26 to mediate repression. The other Bcl11b cofactors discussed here have variably activating and repressive effects on chromatin. The lysine demethylase Kdm1a can demethylate di- or mono-methylated lysines H3K4 and H3K9, resulting in variably active or repressive marks [reviewed in (93)]. The NuRD complex, the most prevalent partner of Bcl11b, uniquely links chromatin remodeling function with histone modification. Its deacetylase subcomplex can recruit HDAC-1 and -2, while the CHD (also known as Mi-2) subunits perform ATP-dependent chromatin remodeling, sliding nucleosomes and in some contexts exchanging H2A for the paralog H2A.Z [reviewed in (94, 95)]. Finally, the positively-acting Swi/Snf complex is an important chromatin remodeling complex, which utilises ATP to physically move nucleosomes along the genome as well, to open regulatory sites in chromatin to facilitate transcriptional activation [reviewed in (96, 97)].

In addition to their impacts on local chromatin states, the chromatin modifying factors with which Bcl11b can be associated have also been closely implicated in regulation of intrachromosomal looping contacts. The Swi/Snf complex has been implicated in mediating higher order interaction in a number of contexts, such as the α- and β-globin loci (98, 99), the Class II transactivator (*Ciita*) locus and in mature T cells in the Th2 cytokine locus (100). Subsequent Hi-C experimentation has allowed researchers to implicate Swi/Snf complexes in loop formation and TAD insulation genome-wide (101). The PRC1 complex has a much-studied role in regulating chromatin looping, a role conserved between *Drosophila* and mammals. Direct binding of PRC1 to genetic regulatory regions is required to establish and maintain chromatin looping in mouse embryonic development (102–106). Recent work in developing neurons has found the Chd4 (Mi-2β) NuRD subunit with chromatin remodeling activity to regulate cohesin binding to enhancer and promoter regions and subsequent TAD boundary strength (107). An important question, then, is whether Bcl11b and its interaction partners only regulate local modifications of chromatin states to favour or disfavour transcription, or if they could indeed be implicated in regulating aspects of 3D chromatin structure over broad domains as well.

#### Bcl11b as a Potential Regulator of Higher-Order Chromatin Structure

The involvement of Bcl11b in remodelling or maintaining chromatin architecture offers an alternative way to explain its impact which does not depend on a simple distinction between target and non-target genes. Over the same developmental transition coinciding with commitment, just as Bcl11b is becoming active, substantial changes occur in the chromatin architecture of developing thymocytes. Whole genome assessment of chromatin accessibility and looping identified the DN2 stage as a key inflection point for the chromatin state of developing thymocytes, with a significant fraction of identified regions flipping in accessibility or A/B compartment identity either side of this developmental stage (42). Hu and colleagues identified Bcl11b bound sites in more developmentally mature CD4+ CD8+ double positive (DP) cells to be significantly enriched for the interactions that were originally established between the DN2 and DP stages. Importantly, previously reported (44) sites of Bcl11b binding in DN3 cells were similarly enriched for topologically associated domains (TADs) that were strengthened between DN2 and DN3 cells (42). These observations provide strong support for a model in which Bcl11b functions to establish and subsequently strengthen much of the chromatin structure of developing thymocytes.

Bcl11b binding domains in mature T cells are associated with distinctive chromatin domain features. Although many of its effects on gene expression are repressive, Bcl11b is most often found binding at open chromatin regions in the cell type being analyzed, with active histone marks (41, 42). Its binding sites appear enriched for anchors of intrachromosomal loops, and loop interactions are reduced when Bcl11b is deleted in mature T cells (42). As described below, there are genomic regions, such as the extended TCRβ coding locus, where the presence of Bcl11b in the cell is needed for effective binding of Runx1 to numerous sites spread over ~200 kb of DNA (40). Thus, it is possible that a major role of Bcl11b is to serve as an adaptor between other sequence-specific factors and non-DNA-binding chromatin modification proteins to define chromatin state boundaries.

Direct evidence that Bcl11b could be a mediator of higher-order chromatin associations, and not just a passenger factor, emerged from conditional depletion in mature T cells. Six days after *Bcl11b* was inactivated with a tamoxifen-inducible Cre *in vivo*, mature, naïve CD4 T cells showed substantial alterations in chromatin looping architecture (42). Notably, no global impact on DNase hypersensitivity was found at Bcl11b-bound sites in this case, suggesting that Bcl11b may be dispensable for maintaining chromatin accessibility of its binding sites in homeostasis. However, consistent with a direct effect of the protein, regions normally bound by Bcl11b displayed significant reductions in intrachromosomal interactions after treatment (42).

In a developmentally significant case, Bcl11b also has an impact on the chromatin state of the TCRβ locus itself that differs from the TCRγ locus (40). Runx1 occupies numerous binding sites at all of the TCR loci, and Runx1 and Runx3 factors are expressed both before and after Bcl11b is turned on (108). However, stage-specific binding patterns and acute *Bcl11b* knockout results show that Runx1 binding at numerous sites across ~150 kb encoding TCRβ locus V regions only occurs when Bcl11b is present, whereas Runx1 binding across the TCRγ locus occurs in the same cells whether Bcl11b is there or not (40). This concerted effect suggests that Bcl11b must co-bind with Runx1 to promote a state switch of this extended TCRβ coding region from chromatin closure to accessibility, and that this may explain the selective defect in TCRβ gene rearrangement in Bcl11b-deficient cells (5). Thus, part of the mechanism enabling the TCRβ to be fully assembled may depend on Bcl11b altering chromatin accessibility across an entire chromatin domain.

Bcl11b may play a more dynamic role in chromatin accessibility when cells are undergoing activation and differentiation. In fact, broad, global changes to the chromatin accessibility landscape of regulatory T (Treg) cells were seen in response to conditional Bcl11b loss (43, 75), and also in Bcl11b-deficient CD4 T cells that developed into Th2 effectors (18), as compared to controls. This supports the idea that Bcl11b has a role in regulating chromatin accessibility, both directly and indirectly.

This role of Bcl11b in maintaining accessibility may be dependent on developmental stage or activation state of the cell. Hu and colleagues saw no impact on accessibility of Bcl11b-bound sites in naïve T cells when *Bcl11b* was deleted (42). On the other hand, when Bcl11b was deleted in Treg cells from thymic development onward, normally Bcl11b-bound Treg signature genes displayed reduced chromatin accessibility. Strikingly however, although many of the same sites were bound by Bcl11b in naïve cells, they did not appear to undergo the same loss of accessibility in naïve CD4 T cells when *Bcl11b* was deleted (43). These contrasts seem to imply a requirement for Bcl11b to regulate chromatin accessibility in developing and differentiating cells, but a dispensability for Bcl11b in this role in homeostasis – though it remains to be seen if this holds true throughout T cell development and differentiation.

Together, these observations provide evidence for a role for Bcl11b in establishing the 3D chromatin architecture of developing thymocytes with an ongoing requirement to maintain chromatin loops, a direct role for Bcl11b in regulating the activity of key T-lineage enhancers, and a varied role for Bcl11b in regulating larger-scale chromatin accessibility.

## Conclusions

Bcl11b is a potent factor, needed to support and repress multiple distinct cellular lineages. In lymphoid development, it can recruit a range of factors and complexes with chromatin-modifying, -remodeling and -looping activities. A recurring theme throughout this review has been the context-specific nature of Bcl11b action: its binding sites, its co-bound proteins and the enhancers it targets are highly dependent upon both the developmental lineage and stage of the cell expressing this protein. And while Bcl11b has demonstrated roles in regulating chromatin structure, there remains much to be elucidated regarding the precise mechanisms involved and their causal impacts on alterations of gene regulation steady states. It is unclear what regulates Bcl11b recruitment to its cognate motif in DNA versus recruitment to pre-bound Runx, ETS or other such transcription factors, or what determines the preference of Bcl11b, such as when protein levels are limiting, for direct versus recruited binding. It further remains to be clarified which domains of Bcl11b recruit a number of its co-bound complexes, and which specifically are recruited by the C-terminal zinc finger for the distinctive set of functions that Bcl11b must exert in mature as opposed to developing T cells and ILC2 cells. Much still remains to be learned too of which of the bound cofactors are responsible for the establishment and maintenance of chromatin modifications and loops by Bcl11b at specific stages in lymphocyte development and differentiation.

The state of the Bcl11b literature to date allows us to conceptualize likely modes of action for Bcl11b that encompass the exceptional context-dependence of its function. Understanding that its diverse chromatin-binding partners and recruited chromatin-modifying complexes are variably expressed throughout T cell ontogeny takes this capriciousness from surprising to inevitable. We look forward to elucidating, alongside others in the field, the direct and co-dependent activities of Bcl11b that drive its diverse functions, and how each is chosen in the appropriate developmental context.

## Author Contributions

TS drafted the manuscript, generated the figures, and wrote the final text. ER edited the manuscript and co-wrote the final text. Both authors approved the submitted version.

## Funding

Relevant work in the Rothenberg group was supported by USPHS grants R01AI083514 and R01AI135200 to ER. ER also received support from the Albert Billings Ruddock Professorship at Caltech.

## Conflict of Interest

ER is a member of the Scientific Advisory Board of Century Therapeutics and has served as Advisor or Consultant for Kite Pharma and A2 Biotherapeutics.

The remaining author declares that the research was conducted in the absence of any commercial or financial relationships that could be construed as a potential conflict of interest.
